# Genetic adaptation as a biological buffer against climate change: Potential and limitations

**DOI:** 10.1111/1749-4877.12298

**Published:** 2018-07-26

**Authors:** Luc De MEESTER, Robby STOKS, Kristien I. BRANS

**Affiliations:** ^1^ Laboratory of Aquatic Ecology, Evolution and Conservation Leuven Belgium; ^2^ Evolutionary Stress Ecology and Ecotoxicology Leuven Belgium

**Keywords:** climate change, evolutionary rescue, evolutionary constraints, evolving metacommunities, extinction risk, range shifts

## Abstract

Climate change profoundly impacts ecosystems and their biota, resulting in range shifts, novel interactions, food web alterations, changed intensities of host—parasite interactions, and extinctions. An increasing number of studies have documented evolutionary changes in traits such as phenology and thermal tolerance. In this opinion paper, we argue that, while evolutionary responses have the potential to provide a buffer against extinctions or range shifts, a number of constraints and complexities blur this simple prediction. First, there are limits to evolutionary potential both in terms of genetic variation and demographic effects, and these limits differ strongly among taxa and populations. Second, there can be costs associated with genetic adaptation, such as a reduced evolutionary potential towards other (human‐induced) environmental stressors or direct fitness costs due to tradeoffs. Third, the differential capacity of taxa to genetically respond to climate change results in novel interactions because different organism groups respond to a different degree with local compared to regional (dispersal and range shift) responses. These complexities result in additional changes in the selection pressures on populations. We conclude that evolution can provide an initial buffer against climate change for some taxa and populations but does not guarantee their survival. It does not necessarily result in reduced extinction risks across the range of taxa in a region or continent. Yet, considering evolution is crucial, as it is likely to strongly change how biota will respond to climate change and will impact which taxa will be the winners or losers at the local, metacommunity and regional scales.

## INTRODUCTION

Climate change is having widespread effects on biota, including range shifts, novel and altered intensities of biotic interactions in changed food webs and host–parasite interactions, and extinctions (Parmesan & Yohe 2003; Parmesan 2006; Gilman *et al.* 2010; Scheffers

*et al.* 2016; Urban *et al.* 2016; McCarty *et al.* 2017). One of the biotic complexities that might strongly influence our predictions of how biota will respond to future climate change (Valladares *et al.* 2014; Urban *et al.* 2016) is the capacity of natural populations to genetically adapt to environmental change (Bradshaw & Holzapfel 2006; Márquez *et al.* 2007; Gienapp *et al.* 2008; Lavergne *et al.* 2010; Hoffmann & Sgrò 2011; Merilä & Hendry 2014; Chirgwin *et al.* 2015). Next to physiological acclimation, altered behavior, and adaptive phenotypic plasticity mediated by maternal effects and epigenetics (Fiedler *et al.* 2004; Fuller *et al.* 2010; Somero 2010; Huey *et al.* 2012; Charmantier & Gienapp 2014; Bell *et al.* 2015; Seebacher & Post 2015; Seebacher *et al.* 2015; Wong & Candolin 2015), evolutionary responses have the potential to provide a buffer against extinctions or range shifts upon climate change (Gienapp *et al.* 2008; Hoffmann & Sgrò 2011; Merilä & Hendry 2014). The emerging insight is that plasticity may not be enough for populations to track ongoing climate change and that genetic changes are needed (Duputié *et al.* 2015; Gunderson & Stillman 2015). While such an evolutionary buffering effect is a possible outcome, in the current opinion paper we argue that there are a number of constraints and complexities that may lead to predictions and outcomes that differ from a mere buffering effect. These may include even stronger range shifts than in the absence of evolution (e.g. in case of evolutionary specialization; Bocedi *et al.* 2013), or profound changes in the distribution, occurrence, or trait values of populations.

In the following, we first provide a short overview of the recent evidence for evolutionary responses to climate change, both in terms of evolution of mean trait values and evolution of plasticity. Second, we discuss constraints on evolutionary change that are likely to limit the capacity of natural populations of many species to respond to climate change by adaptive trait change. These include classic constraints such as the lack of genetic variation or strong genetic correlations among traits that are under contrasting selection pressure, but also costs of evolution at the individual and population level. We consider how the buffering role of evolution in shaping responses to climate change may strongly depend on metacommunity context (see the evolving metacommunity concept in Urban & Skelly 2006; Urban *et al.* 2008). This is important given that climate change and the responses to it have an explicit spatial component through the latitudinal climate gradient determining the direction of range shifts as well as the source of potentially pre-adapted immigrants (Urban *et al.* 2012a). Finally, we make predictions on the conditions in which evolutionary change might impact extinction risk and range shifts in response to climate change. A summarizing conceptual scheme is provided in Fig. 1.

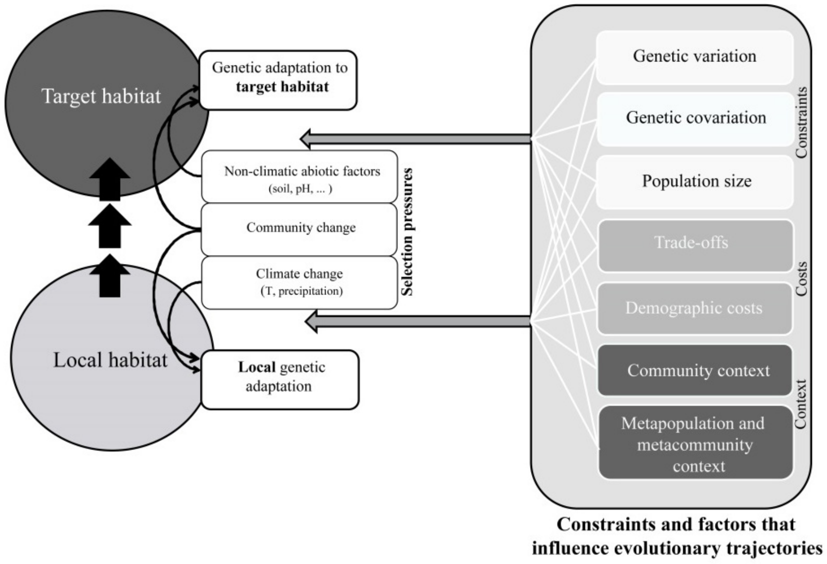

**Figure 1** Simplifying scheme on constraints and factors that impact evolutionary trajectories in response to climate change. Evolution can contribute to responses to climate change both when the population remains in its original habitat as well as when the population migrates to another habitat, although the specific selection pressures will often differ. The degree to which populations can genetically track climate change is dependent on a number of constraints (genetic variation, genetic covariation) that are in part linked to population size, on the costs of evolution both at the level of the population (demographic cost) and in terms of tradeoffs affecting fitness, and on the context provided by the community in which the population is embedded and by landscape features determining metapopulation and metacommunity structure.

## EVIDENCE FOR EVOLUTIONARY RESPONSES TO CLIMATE CHANGE

The capacity of natural populations to genetically adapt to climate change is a topic that is understudied in global change research (Merilä & Hendry 2014). Inspired by the landmark study by Bradshaw and Holzapfel (2001) showing genetic adaptation in phenology of the pitcher plant mosquito in a 5-year time period as growing seasons become longer due to warming, there are an increasing number of studies that document evolutionary changes in phenology (Bradshaw & Holzapfel 2006; Van Asch *et al.* 2007; Merilä & Hendry 2014). Since Bradshaw and Holzapfel (2001), multiple other traits have been shown to evolve in response to climate change, including thermal tolerance, drought resistance and dispersal traits (overviews in Hoffmann & Sgrò 2011; Merilä & Hendry 2014, and the different chapters in that special issue dedicated to phenotypic plasticity and genetic responses to climate change), as well as other traits indirectly related to climate change, such as altered defense responses to antagonistic biotic interactions and host plant choice (Bridle *et al.* 2014; Buckley & Bridle 2014). Overall, genetic responses to climate change have now been reported in a broad range of taxa. Yet, Merilä and Hendry (2014) concluded that acclimation of populations through phenotypic plasticity seems still by far more common than genetic adaptation in mediating responses of natural populations to climate change. Building on these earlier reviews and more recent studies, we here focus on 3 perspectives that may influence our view on how evolutionary change impacts biotic responses to climate change.

First, while the earlier studies on genetic adaptation to climate change predominantly reported changes in phenology, there is also growing evidence for genetic responses in heat tolerance (Huey *et al.* 1991; Weeks *et al.* 2002; Dixon *et al.* 2015; Geerts *et al.* 2015). Recent work on the water flea *Daphnia magna* documented a genetic increase in heat tolerance in response to 4 °C warming in a 2-year experimental evolution trial in outdoor mesocosms (Geerts *et al.* 2015). In addition, resurrection ecology studies demonstrated that natural *D. magna* populations have evolved a higher heat tolerance over the past 40 years (Geerts *et al.* 2015) and a better ability to deal with mild warming within 7 years (Zhang *et al.* 2016). In combination, these studies show that natural populations of *Daphnia* not only have the potential to genetically adapt to future climate change but also have already responded to past climate change over the past decades. Genetic adaptation through a change in heat tolerance may be common, as rapid climate change likely imposes strong selection on this trait, and the trait generally shows heritable variation (Diamond 2017). There are a multitude of studies on terrestrial insects and vertebrates showing genetic changes in heat tolerance across thermal gradients associated with latitude and altitude (Hoffmann *et al.* 2002; Chown *et al.* 2010; Bozinovic & Pörtner 2015; Comte & Olden 2016; Diamond 2017). While such spatial gradients in trait values might often reflect evolution over long time periods, these studies do show that many species currently harbor genetic variation in heat tolerance matching climate gradients. Moreover, Balanya *et al.* (2006) show that clinal latitudinal variation in *Drosophila subobscura* developed in just a few decades, and recent studies showing genetic differentiation in heat tolerance along urbanization gradients tracking temperature change associated with the urban heat island effect similarly suggest that gradients in genetically determined differences in heat tolerance can build up rapidly (Brans *et al.* 2017b; Diamond *et al.* 2017). A transplant study on reef corals also found remarkable genetic adaptation and acclimatization to warming with heat-tolerant populations of tabletop coral *Acropora hyacinthus* showing significantly reduced levels of bleaching upon heat stress within 2 years (Palumbi *et al.* 2014). Mechanisms that may lead to higher heat tolerance involve biochemical adaptation (e.g. heat shock proteins; Tomanek 2010; Bentley *et al.* 2017) and a smaller body size (Daufresne *et al.* 2009; Brans *et al.* 2017a). Evolution of heat tolerance is especially important for species already close to their upper thermal boundaries where plasticity in upper thermal limits is unlikely to effectively buffer effects of global warming (Tewksbury *et al.* 2008; Araújo *et al.* 2013; Sørensen *et al.* 2015; van Heerwaarden *et al.* 2016) and may be enhanced by exposure of hidden genetic variation (Diamond & Martin 2017). Yet, each case will have to be evaluated carefully as the level of heritable variation may not always be enough to deal with predicted warming (Hoffmann *et al.* 2013; Schou *et al.* 2014; Hangartner & Hoffmann 2016). One reason that the evolution of increased heat tolerance could be limited is through a negative relationship with thermal acclimation responses as seen in the dung fly *Sepsis punctum* selected for higher heat tolerance (Esperk *et al.* 2016).

Second, climate change as a selection pressure has many dimensions. While traits linked to heat tolerance, phenology and drought are obvious candidates for studies on genetic adaptation to climate change in many organisms, climate change is indirectly also likely to induce evolutionary change in a broad array of other traits. For example, in the case of a range shift, populations might be confronted with any change in abiotic conditions, such as soil type, pH, landscape structure and chemical pollution. In addition, populations might often be exposed to pronounced changes in biotic interactions, such as a change in prey and host plant availability (Pateman *et al.* 2012), vegetation cover, intensity of competition (Alexander *et al.* 2015), or the presence of predators, parasites or exotic species (Gaedke *et al.* 2010; Gilman *et al.* 2010; Hellmann *et al.* 2012; Shurin *et al.* 2012; Zarnetske *et al.* 2012; Hansson *et al.* 2013) These changes in biotic context may also occur in case populations do not shift ranges, but other species do. For instance, climate change may induce a change in intensity of parasitism and predator–prey interactions (Nussey *et al.* 2005; Hall *et al.* 2006; Durant *et al.* 2007; De Block *et al.* 2013; Bonaviri *et al.* 2017), the abundance of exotic species (Dukes 2010; Wolkovich *et al.* 2013), or a shift in the abundance of host plants (Wolkovich & Cleland 2014). For example, Bridle *et al.* (2014) show that the brown argus butterfly *Aristia agestis* specializes on one host plant species as it expands its range as a consequence of climate warming. In the context of altered predator–prey interactions, increased temperatures typically increase predation rates because of increased attack efficiencies and reduced handling times (Englund *et al.* 2011), and both local evolution and immigration of low-latitude predators may further enhance this effect. For example, two recent studies using a space-for-time substitution approach provided experimental evidence that predation rates by damselfly larvae on water fleas (De Block *et al.* 2013) and on mosquitoes (Tran *et al.* 2016) would increase at high latitude under warming when predators show thermal evolution or warm-adapted low-latitude predators move poleward.

Third, while many studies focus on the dichotomy between phenotypic plasticity and evolutionary change (Merilä 2012; Merilä & Hendry 2014) as buffers against climate change, it is important to also consider that evolutionary change often also involves evolution of plasticity (Brommer *et al.* 2005; Nussey *et al.* 2005; Chown *et al.* 2010; Stoks *et al.* 2016; Chevin & Hoffmann 2017) and that phenotypic plasticity and genetic change can be intrinsically related (Lande 2009; Diamond & Martin 2017). Evolved plastic responses are expected to be important for populations to deal with climate extremes (Kingsolver *et al.* 2007; Chevin & Hoffmann, 2017). For example, Kingsolver *et al.* (2007) showed rapid evolutionary divergence of thermal reaction norms for size, development time and survival in the butterfly *Pieris rapae* when invading North America. In another example, Nussey *et al.* (2005) found heritable variation in individual plasticity in timing of reproduction in great tits and detected past climate change (over a period of 30 years) to have selected for more plastic individuals as a response to climate-driven shifts in peak occurrence of their caterpillar prey.

## CONSTRAINTS ON EVOLUTIONARY CHANGE

### Genetic variation and covariation

In the previous paragraphs, we emphasized that many natural populations have been shown to have the capacity to genetically respond to climate change. The degree to which they can, however, will critically depend on a number of constraints, and several authors (e.g. Jump & Puenelas 2005; Charmantier *et al.* 2008; Merilä 2012; Gienapp *et al.* 2013; Merilä & Hendry 2014; Diamond & Martin 2017) have expressed doubts regarding whether most natural populations will have the capacity to evolve sufficiently fast to keep track with current climate change. Generation time is a key factor, as it determines the speed by which a population can evolve in the face of a given rate of change in the external environment (Réale *et al.* 2003; Visser 2008; Gienapp *et al.* 2013). Small taxa with short generation times will, therefore, all else being equal, in general have a higher capacity to genetically track climate change than species with long generation times, such as mammals (e.g. Boutin & Lane 2014; Hetem *et al.* 2014). Unicellular organisms and many invertebrates might, therefore, be among the organisms for which a considerable capacity for adaptive evolution in response to climate change is to be expected. There are, however, quite a number of examples of contemporary evolution in fish, birds, lizards (Bearhop *et al.* 2005; Crozier *et al.* 2008; Gienapp *et al.* 2008; Jensen *et al.* 2008; Olsson *et al.* 2010; Charmantier & Gienapp 2014; Hendry 2016; Pörtner & Gutt 2016) and even trees (Kremer *et al.* 2012; Alberto *et al.* 2013; Hornoy *et al.* 2015; Yeaman *et al.* 2016).

As a first constraint, the capacity to genetically respond fast enough to climate change critically depends on the presence of a sufficient amount of genetic variation in natural populations, on population size and on genetic variation present regionally (Chown *et al.* 2010; Hoffmann & Sgrò 2011). This constraint can become more important in complex settings in which multiple traits need to evolve simultaneously. Evolution can be fueled by standing genetic variation, by *de novo* mutations or by gene flow (Lynch & Walsh 1998; Barrett & Schluter 2008; Hoffmann & Sgrò 2011). The lack of genotypic variation for traits under selection can cause evolutionary inertia (Bradshaw 1991), which has been observed for a range of taxa in the context of climate change (Hoffmann *et al.* 2013; Kellermann *et al.* 2006; van Heerwaarden *et al.* 2008). For example, 2 rainforest species of *Drosophila* (Kellermann *et al.* 2006) have been shown to lack genetic variation for desiccation resistance, reducing their evolutionary potential to respond to climate shifts. In another example, Chirgwin *et al.* (2015) found adaptive genetic variation for larval survival in 2 cooler temperatures but not in a warmer temperature in the marine polychaete *Caleolaria caespitosa*. At first sight, this suggests that the species would be limited in its evolutionary responses towards warming. Yet, there was additive genetic covariance between larval survival at the coolest and the warmest temperature, and larval survival at each temperature contributed strongly to the multivariate direction of greatest additive genetic variance (gmax) for these 3 traits. Therefore, selection for increased larval survival across different temperatures (in the direction of gmax) should result in an evolutionary increase in survival across all 3 temperatures. This study thus cautions against conclusions that are too simplistic and argues for the need to adopt a multivariate perspective. Indeed, even in the absence of univariate additive genetic variance for survival at the warmer temperature, adaptation in response to warming will still be possible in this example because of the presence of additive genetic variance in multivariate space (survival at the warmer temperature contributes to gmax), and because of the genetic covariance across temperatures (Chirgwin *et al.* 2015).

The amount of standing genetic variation that is maintained in a population depends, among other factors, on effective population size, as effective population size determines the loss of genetic variation due to genetic drift and the likelihood of mutations generating new variants (Hartl & Clark 2007). As a result, all else being equal, we expect genetic responses to climate change to be more prevalent in relatively small organisms that build up large populations. This enhances the dichotomy between small, common species with short generation times and larger, less common species with longer generation times, with the latter often being more constrained in their capacity to genetically respond to climate change. These latter species are also focal taxa in many conservation genetic studies (Frankham 1995; Vander Wal *et al.* 2013). Even huge population sizes are, however, no guarantee for evolutionary rescue. For example, Kwiatkowkski & Roff (1976) documented in the 1970s that phytoplankton populations estimated to be up to $10^{10}$ cells per lake failed to adapt to acidification caused by plume pollution coming from a nickel smelter. In one case, a community of 55 species was reduced to only one *Chlorella* population. This population eventually did evolve resistance and was capable of persisting through 20 years of gradual acidification.

In addition to local population sizes, genetic variation present at the regional, landscape context may also play a key role in adaptation to climate change. Adaptive evolution of a local population may, indeed, be fueled by gene flow from pre-adapted populations and cause "genetic rescue" (Aitken & Whitlock 2013; Gomulkiewicz & Shaw 2013; Carlson *et al.* 2014). This may result in a replacement of one population of a given species by another one, or result in rapid dominance of an immigrant, advantageous allele through a selective sweep (Carlson *et al.* 2014; Wilson *et al.* 2017). While ecologists would observe no change in the occurrence of a given species over vast areas of its range, this might conceal a replacement at the landscape genetic level.

An interesting complexity in the context of climate change is that genetic diversity and composition can be different in marginal compared to central populations (Eckert *et al.* 2008). In marginal populations, genetic diversity is often lower and genetic differentiation among populations enhanced (e.g. Swaegers *et al.* 2015; Ursenbacher *et al.* 2015), and in areas or range expansion rare variants can increase in frequency due to gene surfing (Excoffier *et al.* 2009). These processes can reduce evolutionary potential of marginal populations, as was shown for the damselfly *Ischnura senegaliensis* (Takahashi *et al.* 2016). Conversely, glacial refugia often are hotspots of genetic diversity whose protection might be crucial for safeguarding evolutionary potential in the face of climate change (Razgour *et al.* 2013).

A second constraint results from strong genetic correlations between traits that are subject to conflicting selection pressures (Etterson & Shaw 2001; Duputié *et al.* 2012; Merilä 2012; Chevin 2013). Such genetic correlations can be due to pleiotropy (i.e. when the same loci influence multiple traits) or linkage disequilibrium (i.e. if the traits are influenced by different loci but selection, physical linkage or other evolutionary forces maintain a non-random association between the alleles at these loci; Lynch & Walsh 1998; Conner 2002; Chevin 2013). Genetic correlations can strongly impact the rate of evolution and influence evolutionary trajectories (Berger *et al.* 2013; Careau *et al.* 2015). Yet, few examples exist in the context of climate change. A notable exception is the study by Etterson & Shaw (2001, 2012) showing that, although genetic variance for physiological and morphological drought-related traits was present in 3 populations of North American prairie plants, limited adaptive evolution was predicted in response to increased temperatures and aridity due to strong among-trait antagonistic genetic correlations. In contrast, some studies have shown that genetic covariances can be overcome through strong selection (Frankino *et al.* 2005; Agrawal & Stinchcombe 2009; Conner *et al.* 2011) and it is still not clear how important genetic correlations are in slowing down adaptation in response to climate change.

## Costs of evolution at the population level

If there is sufficient genetic variation for adaptive evolution tracking climate change, this may lead to evolutionary rescue (i.e. survival of the population or species thanks to evolution) (Gomulkiewicz & Holt 1995; Bell & Gonzalez 2009; Gonzalez *et al.* 2013; Vander Wal *et al.* 2013; Carlson *et al.* 2014). Adaptation has, however, a demographic cost (Haldane 1957; Bell 2013; Gomulkiewicz & Shaw 2013), and it is possible that evolutionary change reduces population sizes to such an extent that the population becomes at risk of extinction (Gomulkiewicz & Holt 1995). Another cost of evolution at the population level is reduction of genetic variation. If the population goes through a population bottleneck because of the demographic cost of evolution, genetic drift may lower genetic variation at the genome-wide level, reducing the capacity of the resulting population to genetically respond to novel stressors (Via & West 2008; Via 2009; Hoffmann & Sgrò 2011; Pauls *et al.* 2013). In addition, evolution may also reduce genetic variation in the traits that are subject to selection (e.g. body size and thermal tolerance) and the genomic regions that hitch-hike along, reducing genetic variation in specific regions of the genome (Ferriere & Legendre 2013; Pauls *et al.* 2013). For example, in laboratory experiments using nonbiting midges it was found that exposure to a stressor over a few generations lowered genetic diversity by adaptation as well as by enhanced genetic drift (Vogt *et al.* 2007; Nowak *et al.* 2010) and reduced the possibility of the populations to adapt to a secondary stressor (Vogt *et al.* 2010). Overall, one can expect that small-bodied species with large population sizes are less likely to be strongly impacted by these processes than species that are large ecological specialists or endangered, and, hence, have relatively small population sizes (Charlesworth *et al.* 1997). For instance, Orsini *et al.* (2016) did not observe a reduction in genetic diversity in populations of the water flea *Daphnia* following exposure to pronounced and well-documented environmental changes in their analysis of SNP variation in layered dormant egg banks, even though the populations had been documented to have genetically adapted to these environmental changes.

In cases where gene flow fuels adaptive genetic change in local populations, this entails 3 risks in the context of adaptation and performance. First, gene flow between 2 populations can lead to outbreeding depression when populations have been separated for a long time (Frankham 1995). Local adaptation to important non-climatic environmental factors (e.g. soil conditions) that differ between source and target habitats might cause maladaptation after migration, leaving populations vulnerable in the light of climate change adaptation (Aitken & Whitlock 2013). Second, if immigrant genotypes are disproportionally successful in relatively small local populations so that their offspring dominates the populations, this might lead to overall genetic impoverishment (Via & West 2008; Via 2009; Pauls *et al.* 2013). Third, even in larger populations where recombination leads to an efficient selection on the beneficial, immigrant allele, the resulting selective sweep lowers genetic variation at the selected locus and linked genomic regions. “Soft” selective sweeps, in which multiple adaptive lineages contribute to the initial evolutionary rescue, are, therefore, more likely to drive long-term population persistence through evolution (Wilson *et al.* 2017).

## Costs of evolution at the individual level: Tradeoffs

Evolutionary trait change in response to natural selection can have costs linked to tradeoffs (Clarke 2003;

Chaianunporn & Hovestadt 2015; Chirgwin *et al.* 2015; Kelly *et al.* 2016; Sørensen *et al.* 2016). For example, molecular chaperones such as heat-shock proteins are costly to produce and can lead to reduced somatic growth, developmental rate and fertility (Silbermann & Tatar 2000; Sørensen *et al.* 2003). Kelly *et al.* (2016) report that experimental evolution of increased heat tolerance resulted in a lower fecundity in the intertidal copepod *Tigriopus californicus*. In another example, Hughes *et al.* (2003) showed that climate-driven range expansion in the speckled wood butterfly (*Pararge aegeria*) shows a strong association with evolutionary changes in dispersal capacity, but also comes at a cost of reduced reproductive investment. Such tradeoffs and fitness costs towards responses to other stressors can result because of a direct conflict at the trait level (e.g. a reduction in body size to increase thermal tolerance according to the temperature-size rule makes individuals more susceptible to a gape-limited predator) or can result from a reduced energy allocation to other traits such as chemical tolerance, anti-predator defenses or parasite immunity (Clarke 2003). For example, a tradeoff between thermal adaptation and tolerance to pollutants (Moe *et al.* 2013) was recently documented across latitudes with warm-adapted low-latitude damselflies being more vulnerable to the trace metal zinc than cold-adapted high-latitude damselflies (Debecker *et al.* 2017). In addition to tradeoffs, costs of evolution can also result from pleiotropy (i.e. genes impacting different traits) or linkage. At the same time, while tradeoffs or other costs might be common, they are not necessarily always present. For instance, Hangartner and Hoffmann (2016) could not detect a cost in terms of vulnerability to predation by jumping spiders after experimental evolution of increased heat tolerance in the fruitfly *Drosophila melanogaster*.

In the context of climate change, these indirect fitness costs of evolution towards responses to other stressors likely have important consequences, precisely because climate change is often associated with pronounced changes in other factors such as biotic interactions (Post 2003; Parmesan 2006; Both *et al.* 2008; Gilman *et al.* 2010; Urban *et al.* 2016) and increased pollution levels (Kattwinkel *et al.* 2011). In addition, climate change is only one aspect of global change, and many populations are additionally impacted by other human-induced stressors such as pollution, eutrophication, exotic species and urbanization (Palumbi 2001; Grimm *et al.* 2008; Butchart *et al.* 2010; Alberti *et al.* 2017). Indirect fitness costs of adaptive evolution in response to climate change warrant attention and might strongly impact our predictions on the impact of climate change.

## GENETIC ADAPTATION IN A METACOMMUNITY CONTEXT: LOCAL VERSUS REGIONAL RESPONSES

As there is an intrinsic spatial component to responses to climate change, it is important to include metapopulation and metacommunity-level dynamics when studying those responses. As populations may genetically adapt to climate change, the evolving metacommunity concept provides a strong framework to consider the interactions between community composition, evolutionary trait change, the environment and space (Urban *et al.* 2008, 2012; De Meester *et al.* 2011). The potential for interactions between ecological and evolutionary responses (i.e. "eco-evolutionary dynamics"; Hendry 2016) is very rich and complex, but as a first approach we can focus on two questions. First, what is the relative importance of local and regional dynamics in the responses to climate change? Second, what is the relative importance of evolutionary and ecological dynamics in the responses to climate change?

Evolution can impact responses to climate change in three ways, which we briefly discuss in the following paragraphs. First, local genetic tracking of climate change can allow populations to survive locally and, thus, make responses to climate change more local (i.e. reduce the extent of range shifts). Second, when populations migrate along with the moving climate, genetic adaptation to the novel conditions they encounter might contribute to survival in the newly colonized areas, thus impacting effective range shifts. Third, evolution of dispersal can impact the speed and extent of range shifts.

### Evolutionary change can make responses more local

Traditional climate envelope models predict vast changes in distribution patterns of organisms in response to climate change (Bakkenes *et al.* 2002; Thuiller 2003; Brooker *et al.* 2007; Chen *et al.* 2011; Su *et al.* 2015). These predictions might in some cases, however, be impacted by assumptions underlying the models, which often do not take dispersal limitation or complex dispersal dynamics, nor evolution into account (Urban *et al.* 2013, 2016; Weiss-Lehman *et al.* 2017). If populations can locally track climate change through evolution, they might not be forced to shift their range. In practice, when local populations are confronted with climate change, they will respond in both ways: a subset of individuals will migrate and might colonize other patches where the climate is similar to that in their former patch, whereas others will remain and might respond to the changing selection pressures by genetic trait change. Depending on the success of these strategies, the result might be anything from range shift, range expansion, range contraction, to extinction. The outcome of these parallel strategies will for each population depend on a number of factors, such as the capacity for (long-distance) dispersal and the evolutionary potential of local populations. Some interactions between the capacity to disperse and the capacity to evolve can be expected, such as the prediction that local genetic adaptation is more difficult in populations that are exposed to high levels of gene flow (Lenormand 2002; Kinnison & Hairston 2007; Bridle *et al.* 2010; Bourne *et al.* 2014). Yet, there are multiple mechanisms through which local genetic adaptation in the face of high dispersal is possible (i.e. "microgeographic adaptation"; Richardson *et al*. 2014). Species with low dispersal capacity and low capacity to evolve (e.g. large-bodied species with long generation times and low population sizes; Hoffmann *et al.* 2017) are most at risk of extinction. Species with high dispersal capacity but low evolutionary potential (e.g. birds or butterflies with relatively long generation times) can be predicted to show pronounced range shifts. If the habitats or communities they encounter as they shift ranges are very different, however, this might result in strong mismatches and local extinction (Schiffers *et al.* 2013). Species with low dispersal capacity and high evolutionary potential are expected to show local responses and might be able to genetically track climate change. Species with high dispersal capacity and high evolutionary potential might both expand their niche and locally adapt. The actual responses will, however, also strongly depend on context, such as the density and abundance of antagonists in the expansion zone, or the level of competition imposed by immigrants for the populations that stay resident. In addition, as different competing species can differ in the degree to which they show local or regional responses, and this can lead to conflicts and extinction (De Meester *et al.* 2011; Urban *et al.* 2012a). Indeed, if species with adjacent ranges differ in the degree to which they respond by migration or local adaptation, their ranges might increase in overlap and competitive exclusion might occur (Urban *et al.* 2012a,b).

Species with high evolutionary potential and good dispersal capacities might be the winners of climate change and might be the only species that can profit from the global human-induced changes in environmental conditions, together with species that exhibit high tolerance at the individual level (Williams *et al.* 2008; McGill *et al.* 2015). The opportunistic species that contribute to the homogenization of communities in a human-dominated world (Vitousek *et al.* 1997; McKinney & Lockwood 1999; McKinney 2006)) might be either opportunistic because of high individual versatility or because of a combination of high evolutionary potential and short generation times, conferring versatility at the population level.

## Range shifts and evolutionary adaptation to novel habitats

Natural settings are complex, and a factor that is often overlooked in both modeling and empirical studies on climate change responses is that climate is not the only selection pressure impacting local populations. Temperature is a key environmental factor profoundly influencing metabolism and all physiological processes of organisms (Pörtner & Knust 2007; Tewksbury *et al.* 2008; Angilletta 2009) and their occurrence in time and space (Parmesan & Yohe 2003). Yet, one may argue that temperature and precipitation levels might often be the only abiotic factors that are changing in the local setting, while many other abiotic (e.g. soil type, pH and salinity) and biotic conditions might change if organisms move to other areas. Moreover, given that latitudinal isoclines for summer and winter temperatures strongly differ (Bradshaw & Holzapfel 2006), species moving poleward cannot simply follow their thermal envelope for both summer and winter. It is, therefore, somewhat naïve to assume that dispersal would free organisms from having to evolve. Whether staying in the local habitat or migrating to other patches will lead to the largest changes in selection pressures will depend on the degree of climate change, the amplitude and grain of environmental variation in the landscape, the type of gradients in the landscape (i.e. whether the environmental variables that change are important niche axes of the species), and the degree of changes in biotic selection pressures (competitors, parasites, predators, mutualists; exotic species) that the population will be exposed to locally or in the target region.

If range shifts involve colonization of areas that are free of competitors (e.g. as in areas with retracting permafrost or ice sheets; tree line shifts), this can result in selection for traits that are typical for pioneer species, such as fast intrinsic growth rates (Williams *et al.* 2008). In addition, the abundance of resources combined with colonization by a low number of individuals may result in gene surfing (Excoffier *et al.* 2009) and priority effects, which may lead to reduced genetic diversity within and increased genetic differentiation among marginal habitats (Swaegers *et al.* 2015; Ursenbacher *et al.* 2015). These peculiar genetic characteristics of marginal populations might strongly impact the direction of evolutionary trajectories and the rates of adaptation.

### Evolution of dispersal associated with range shifts

Evolution of dispersal capacity itself might also strongly impact responses to climate change in those species that show a range shift (Kubisch *et al.* 2014). Models (Phillips *et al.* 2008), field data (Phillips *et al.* 2006) and an increasing number of experiments (Fronhofer & Altermatt 2015; Williams *et al.* 2016; Weiss-Lehman *et al.* 2017) have provided evidence that dispersal can evolve as species expand their range. One mechanism that leads to an increase in dispersal capacity as species expand their range is spatial sorting (Lindström *et al.* 2013). In spatial sorting, genotypes with higher dispersal capacity are automatically promoted at the margin of an expanding range, not because they have a higher fitness in the colonized habitats but because they move faster and there is available habitat. The impact of evolution of increased dispersal rates on the rate of range shifts is considerable (Phillips *et al.* 2006; Fronhofer & Altermatt 2015). High dispersal rates at range fronts might even cause organisms to move faster than the moving climate envelope. Such high dispersal rates from central populations to the expanding range front may explain the observed evolutionary change to reduced heat tolerance and increased winter survival in populations at the range front in the poleward moving wasp spider *Argiope bruennichi* (Krehenwinkel & Tautz 2013; Krehenwinkel *et al.* 2015).

## RESPONSES TO CLIMATE CHANGE IN A METACOMMUNITY CONTEXT: INTERSPECIFIC AND INTRASPECIFIC RESPONSES

From the perspective of the trait distribution of a local community, both shifts in community composition and evolutionary responses within the populations of community members can contribute to a change in trait distribution upon environmental change (Bolnick *et al.* 2011; Violle *et al.* 2012). For example, an increase in temperature might lead to a shift of the community-level temperature performance optimum. This can be mediated by: (i) genetic adaptation of member species that track the change in temperature; (ii) changes in the relative abundance of species that were already present in the community towards increased relative abundances of species with higher temperature optima; or (iii) differential extinction of local species and colonization by species adapted to higher temperatures from the regional species pool. Identifying the relative importance of intraspecific and interspecific contributions to trait change under climate change is, therefore, important (Violle *et al.* 2012; Govaert *et al.* 2016). While the capacity of local populations to genetically adapt depends on their evolutionary potential and their exposure to maladaptive gene flow (Richardson *et al.* 2014), the likelihood of rapid changes in species composition depends on local community species richness, connectedness of the local community through dispersal, and species richness in the metacommunity (cf. metacommunity context; Leibold *et al.* 2004). The capacity to evolve and the scope for rapid changes in community composition are not necessarily related, and this might lead to complex interactions. In regionally species-poor communities with species having low evolutionary potential, the capacity of local communities to adjust to climate change is low. In regionally species-rich communities with species having low evolutionary potential, local communities will primarily respond by changes in the relative abundance of species. In regionally species-poor communities with species having high evolutionary potential, it is in principle possible that the community adjusts to climate change through evolution of all its member species, with community composition staying unaltered. Finally, in regionally species-rich communities with species having high evolutionary potential, the type of response observed will depend on the race between adaptation and immigration (De Meester *et al.* 2016; Vanoverbeke *et al.* 2016). As evolutionary change takes time, local populations can at any moment be replaced by immigrants of pre-adapted species from the metacommunity, even when they have the capacity to locally adapt (Vanoverbeke *et al.* 2016). Whether this will happen will depend on how fast evolutionary adaptation proceeds: if the local population can be sufficiently adapted to the changed environment so that its fitness is higher than the immigrant species, it might prevent establishment of the invaders (Van Doorslaer *et al.* 2009; De Meester *et al.* 2016). Therefore, we predict that evolutionary adaptation of local species to climate change might be reduced in settings with high local species diversity as well as in strongly connected local communities in species-rich metacommunities. In these scenarios, we predict that responses to climate change will largely be mediated by changes in species composition (i.e. community ecology), and less by evolutionary responses to climate. This does not imply the absence of evolution, as the interacting populations might still evolve in response to changes in biotic interactions. Yet, the change in community-level distributions of trait values directly linked to climate is expected to be mediated by changes in species composition rather than by evolution. This leads to the companion prediction that evolutionary responses to climate change will be more important in species-poor local communities that are somewhat isolated (De Meester *et al.* 2016).

## A FEW HINTS AT PREDICTIONS AND IMPLICATIONS

### Contexts that facilitate evolutionary change in response to climate change

From the above, some predictions can be derived on the settings in which evolution might play an important role in the response to climate change. We predict that evolutionary tracking of climate change will be more important in: (i) small-bodied species with short generation times and large effective population sizes; (ii) in species that have a high evolutionary potential relative to their capacity to disperse, allowing sufficient time for local populations to adapt before they would be replaced by pre-adapted individuals from other populations in the metapopulation (see De Meester *et al.* 2016); and (iii) in populations that inhabit species-poor communities in relatively isolated habitats, so that the response to climate change is not dominated by changes in species composition. This applies to communities on islands or inhabiting relatively rare habitat types, but also leads to the prediction that human-induced habitat fragmentation and loss in biodiversity might lead to an increase in the importance of evolutionary change (De Meester *et al.* 2016).

### Climate change, evolution and the risk of local and regional extinction

Evolutionary change can buffer populations locally in the face of climate change, next to high phenotypic plasticity in physiology or behavior, and epigenetic responses. It does, however, not necessarily prevent extinction. As mentioned above, evolution can in some cases directly contribute to extinction through a reduction in population size and an associated increase in demographic stochasticity (Gomulkiewicz & Holt 1995; Orr & Unckless 2008, 2014). Next, genetic adaptation to the stressors associated with climate change may have negative consequences on the capacity of the individuals to deal with other stressors (Van Straalen 2003). In addition, differences in the degree to which competing species respond through local genetic tracking or through migration can result in increased antagonistic interactions and competitive exclusion (Urban *et al.* 2012a,b). It is, therefore, not necessarily so that evolution will lead to a major reduction in population and species extinctions under climate change. This does not imply that evolution will be unimportant. Rather, we can predict that evolution is likely to profoundly change the dynamics of populations and species in response to climate change but not necessarily lead to evolutionary rescue (Urban *et al.* 2016).

### Climate change, evolution and assisted migration

What are the consequences of the above perspectives with respect to the much debated idea of assisted migration as a management tool to safeguard species and ecosystem services in the face of climate change (McLachlan *et al.* 2007; Hoegh-Guldberg *et al.* 2008; Richardson *et al.* 2009; Pedlar *et al.* 2012; Lunt *et al.* 2013)? Assisted migration may be needed in some cases to prevent extinction: for example, in populations that live in strongly fragmented systems, islands or isolated patches and do not have the capacity to acclimatize or genetically adapt to climate change (McLachlan *et al.* 2007). However, assisted migration should not be implemented too lightly, as it might also strongly perturb spontaneous recovery. It may, for instance, reduce the capacity for genetic adaptation of residents in the target habitats, as they are confronted with potentially pre-adapted immigrants (i.e. the race between adaptation and immigration in Vanoverbeke *et al.* 2016). The success of assisted migration will also depend on the capacity of the translocated population to deal (through acclimatization or genetic adaptation) with the novel environmental conditions in the target area (Pelini *et al.* 2009). In addition, there is a risk, by perturbing relationships with predators, parasites and competitors, that translocated populations become invasive (Mueller & Hellmann 2008). When assisted migration is applied to species at risk of extinction, these risks might be low. One should, however, carefully assess risks in case one would like to apply assisted migration as a recurrent and widely used management tool (Schwartz *et al.* 2012).

Assisted gene flow (i.e. gene flow that is actively mediated by humans; McLachlan *et al.* 2007; Hunter *et al.* 2007) to boost population persistence through the introduction of added genetic variation might help populations to survive locally. In addition, assisted gene flow might boost the rate of evolutionary change, which might prevent replacement by well-adapted immigrants (De Meester *et al.* 2016). However, the above considerations lead to the prediction that this will not always guarantee evolutionary rescue. At the same time, assisted gene flow might entail risks, such as disturbance of landscape genetic structure (if a local population is swamped by conspecific immigrants that were pre-adapted while it had the capacity to locally adapt; De Meester *et al.* 2016) and outbreeding depression (if immigrants and residents have adapted to other ecologies, both in terms of biotic interactions or of adaptation to environmental gradients; Frankham 2005; Rhymer & Simberloff 1996). While it might be necessary under some conditions, assisted gene flow should not be implemented lightly and without consideration of the risks involved.

### Evolutionary change and predictions of range shifts and biological responses to climate change

Although evolution might not guarantee a buffer against extinction, it is clear from the above that taking evolution into account will profoundly change our predictions on how biota will respond to climate change (Urban *et al.* 2016) and can inform management (Lankau 2007; Faith *et al.* 2010; Carroll *et al.* 2014; Sarrazin & Lecomte 2016). Some of the differences in predictions are far-reaching, such as the degree to which species will or will not show range shifts, the extent of the range shifts, and the number and identity of species that will go extinct. This is why Urban *et al.* (2016) make a plea to implement evolutionary potential and other biological features (demography, species interactions, dispersal and physiology) into mechanistic models to generate more realistic predictions of biological responses to climate change. In addition, similar to the fact that second-order effects of climate change may induce a whole array of additional evolutionary responses (including co-evolution with novel predators, parasites and mutualists), it has been shown that evolution may also feedback on a whole array of ecological dynamics (i.e. eco-evolutionary dynamics; see Hendry 2016). For example, if a given species can survive locally thanks to evolution, it might impede establishment success of a competitor, and this might, in turn, reduce the success of a parasite. Ignoring evolution in our predictions of climate change responses might, thus, lead to erroneous predictions also in terms of community and ecosystem structure, food web interactions, energy transfer functions and ecosystem services (Urban *et al.* 2016).

## ACKNOWLEDGMENTS

LDM thanks Ray Huey, Lauren Buckley and Wei-Guo Du for the invitation to speak on the symposium “Biological buffers and the impacts of climate change.” We thank an anonymous reviewer for valuable comments on an earlier version of our manuscript. This research was funded by KU Leuven Research Council project funding PF/2010/007 and C16/17/002, BELSPO IAP project SPEEDY, and the Fund for Scientific Research-Flanders (FWO projects G052417N and G0B9818N, and FWO network EVENET).

**Cite this article as:**

## Cite this article as:

De Meester L, Stoks R, Brans KI (2018). Genetic adaptation as a biological buffer against climate change: Potential and limitations. *Integrative Zoology* **13**, 372–91.
